# Magnetic resonance imaging detection of multiple ischemic injury produced in an adult rat model of minor stroke followed by mild transient cerebral ischemia

**DOI:** 10.1007/s10334-016-0597-5

**Published:** 2016-11-04

**Authors:** Ursula I. Tuor, Min Qiao

**Affiliations:** Department of Physiology and Pharmacology, Hotchkiss Brain Institute, Faculty of Medicine, University of Calgary, 3280 Hospital Dr, NW, Calgary, T2N 2T8 Canada

**Keywords:** Ischemic attack, Transient, Magnetic resonance imaging, Animals, Cell death, Stroke

## Abstract

**Objectives:**

To determine whether cumulative brain damage produced adjacent to a minor stroke that is followed by a mild transient ischemia is detectable with MRI and histology, and whether acute or chronic recovery between insults influences this damage.

**Materials and methods:**

A minor photothrombotic (PT) stroke was followed acutely (1–2 days) or chronically (7 days) by a mild transient middle cerebral artery occlusion (tMCAO). MRI was performed after each insult, followed by final histology.

**Results:**

The initial PT produced small hyperintense T_2_ and DW infarct lesions and peri-lesion regions of scattered necrosis and modestly increased T_2_. Following tMCAO, in a slice and a region adjacent to the PT, a region of T_2_ augmentation was observed when recovery between insults was acute but not chronic. Within the PT slice, a modest region of exacerbated T_2_ change proximate to the PT was also observed in the chronic group. Corresponding histological changes within regions of augmented T_2_ included increased vacuolation and cell death.

**Conclusion:**

Within regions adjacent to an experimental minor stroke, a recurrence of a mild transient cerebral ischemia augmented T_2_ above increases produced by tMCAO alone, reflecting increased damage in this region. Exacerbation appeared broader with acute versus chronic recovery between insults.

**Electronic supplementary material:**

The online version of this article (doi:10.1007/s10334-016-0597-5) contains supplementary material, which is available to authorized users.

## Introduction

Stroke is a leading cause of death and disability, with mini-strokes being important warning signs of increased risk for a recurrent stroke. Studies have reported that 15–26% of patients with a recent ischemic stroke have a history of a transient ischemic attack (TIA), with nearly half (43%) occurring in the week prior to the stroke [1]. Following a transient ischemic attack or minor stroke, the risk of a recurrent stroke is reported to be ~2.5–3.1% within 1–2 days, 5.3–5.6% within 1 week, and 7.1–11.2% at 90 days [2, 3]. Of TIA patients with a recurrent event, 53% have been reported to become disabled [4]. Thus, an improved understanding of how multiple injuries combine and their dependence on timing between insults is important, yet difficult to study, in part because there are few simple animal models of recurrent stroke available, and assessing cumulative brain damage following mild ischemic insults is challenging.

One study in the rat indicates that the ensuing damage produced by a recurrent model of transient ischemic attack (multiple mild cortical ischemia) is influenced by the recovery time between them [5]. In this study, histological outcomes assessed damage due to a transient middle cerebral artery occlusion (tMCAO) of sufficient severity to produce diffuse cortical selective cell death, and when repeated, resulted in greater brain damage if recovery prior to the second insult was acute (1 day) versus subacute (3 days) [5]. One disadvantage of using this multiple tMCAO model is that a mild tMCAO does not mimic well a minor stroke or a TIA that is associated with small infarcts, which occur in ~34–39% of TIA patients that have diffusion-weighted imaging (DWI)-detected lesions [6, 7]. A better understanding of the pathophysiology of multiple ischemic events that involve minor strokes followed by tMCAO is also important, considering that an increasing number of methods for recanalization of MCAO include the use of TPA (tissue plasminogen activator) and/or endovascular reperfusion therapy [8–10]. Because minor strokes produced by reperfusion likely contain an infarct core, with a peri-infarct region of milder injury [11, 12], the present study was designed to assess the interactions of a minor stroke and its penumbra with a mild tMCAO mimicking a transient ischemic attack. The minor stroke was created using our photothrombosis (PT) procedure [13] that easily produces small infarcts with a peri-lesion area of mild ischemic damage. Because mini- or minor infarcts are generated, this models most closely a lacunar stroke or small MCA distal artery occlusion. Another advantage of using minor PT is that prior to the second ischemia, the location and severity of the first insult can be identified using magnetic resonance imaging (MRI) [13].

Although standard MRI does not necessarily detect the selective cell death associated with transient mild cerebral ischemia [12, 14], we hypothesized that standard T_2_ MRI would detect ischemic injury near a minor stroke with peri-infarct scattered necrosis, and that following a second insult (tMCAO) [5], cumulative damage in peri-lesion regions would be detected directly using repeated MRI in the same animal. Timing between insults was selected to be 1–2 (acute) or 7 days (chronic), thereby investigating recurrent effects at an early period when recurrence is high and at a longer 1-week recovery—a time that has not been investigated previously.

## Materials and methods

### Experimental animals

**Fig. 1:**
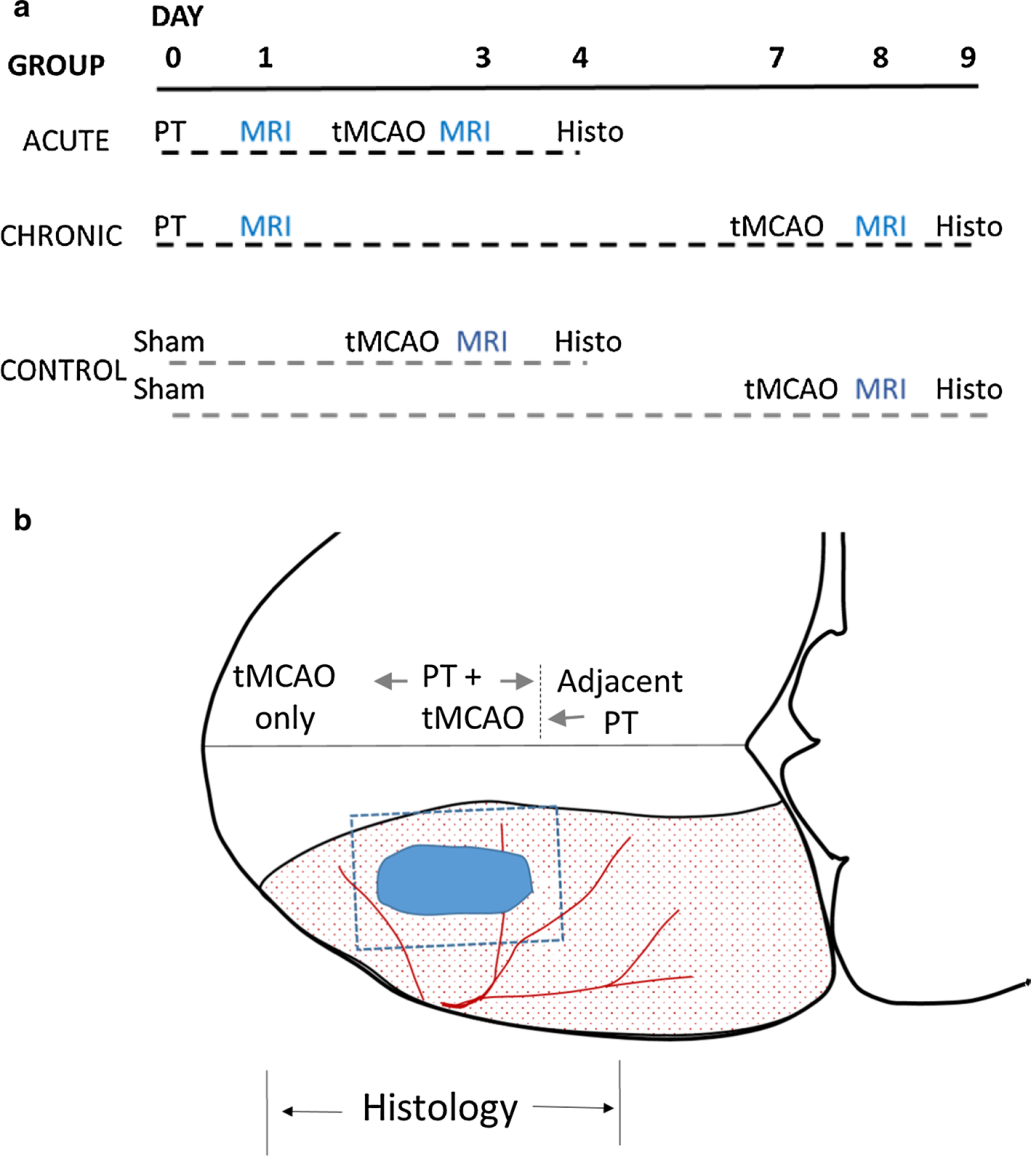
Experimental design. **a** Rats were randomly assigned to acute, chronic, or control groups. Acute and chronic groups were subjected to a photothrombotic (PT) stroke followed by acute or chronic recovery prior to a transient middle cerebral artery occlusion (tMCAO). MRI imaging was performed after each insult, followed by final histology (Histo). A sham control group had a sham PT procedure followed by tMCAO at either 1 or 7 days. **b** Damage was assessed within three levels containing a PT lesion (*solid*) or MCA territory (*stippled*). Levels included an MR slice with both a PT + tMCAO insult, a slice adjacent to the PT, and a slice within the middle cerebral artery territory alone

Experiments were performed using rat models of minor stroke and transient ischemic attack in which the timing of insult was varied (Fig. 1a). Young adult male Wistar rats (Charles River, Montreal, Canada) were randomly assigned to groups of six each. The effects of combined ischemic insults were investigated by comparing responses in the acute group undergoing PT surgery followed by tMCAO 1–2 days later with those in a chronic group with PT surgery followed by transient MCAO 7 days later. The lack of effect of PT surgery on tMCAO was confirmed in a sham control group with PT surgery (saline administration) followed by tMCAO at an acute or chronic time post-sham (*n* = 3 each).

### Production of a minor photothrombotic lesion

Minor photothrombotic ischemic lesions were produced in the parietal cortex using aseptic methods as described previously [13]. Briefly, animals were anesthetized with isoflurane (2–2.5%), and normothermia was maintained at a rectal temperature of 37.0–38.0 °C. The scalp was retracted, and the skull was exposed and thinned to opacity using a saline cooled drill. A mask (3 mm × 5 mm) was positioned on the skull to illuminate the parietal cortex within the middle cerebral artery territory with a cold white light having a beam of maximal intensity centered near the sensory motor cortex. Rose Bengal, a photoactivatable dye generating coagulation, was administered intravenously (10 mg/kg). One minute following its injection, the skull was illuminated for 5 min. Surgical sites were then closed with 3-0 nylon sutures, and buprenorphine (0.03 mg/kg) was administered to provide analgesia.

### Production of transient mild ischemia

A transient ischemic insult was produced in isoflurane-anesthetized rats by temporarily occluding the distal MCA for 30 min using a microaneurysm clip, along with concurrent transient occlusion of both common carotid arteries as described previously [5, 14]. Rectal temperature was maintained using a servo-controlled heating lamp. Arterial blood samples were obtained from a tail artery, and laser Doppler flowmetry was measured through a small burr hole over the cortex. Buprenorphine (0.03 mg/kg, s.c.) provided analgesia. Following surgery, rats were housed in separate cages with free access to soft and hard food, water, and environmental enrichment.

### Magnetic resonance imaging

MR scans were acquired 24 h after ischemic insults using a 9.4T 20-cm-diameter bore horizontal magnet, a Bruker BGA-12S gradient system with nine room temperature shims, and a Bruker Bio-Spin MR imaging system with ParaVision 5.1 software. Rats were anesthetized with isoflurane (1.5–2.5%), and respiration was monitored and temperature was controlled. The rat’s head was secured using ear pins in a 35-mm quadrature volume radiofrequency coil positioned in the center of the bore of the magnet. A set of three orthogonal high-resolution gradient echo scans (TR = 100 ms, TE = 4 ms, flip angle 20 °) were initially acquired, and anatomical features of the cerebellum and ventricles were used to position slices at the same location between scanning sessions. Coronal slice registration between sessions was verified using alignment of white matter and ventricular structures. Images were then acquired with a 3 cm × 3 cm field of view for 20 0.7-mm-thick contiguous slices covering the cerebrum. A multi-slice multi-echo (MSME) spin-echo method with a 32-echo train and 10-ms echo spacing was acquired (TR = 7000, matrix size of 96 × 128 reconstructed to 128 × 128). Bruker software was used to calculate T_2_ maps. Echo-planar diffusion-weighted images gated to respiration were acquired using five *b* values (74.5, 292, 655, 891, and 1164 s/mm^2^), a TR of 5000 ms, an echo time of 40 ms, and a matrix size of 128 × 128. The diffusion images 24-h post-PT displayed areas containing well-defined borders of DW hyperintensity that were used to quantify ischemic lesion areas of infarction [13]. Quantitation of the apparent diffusion coefficient (ADC) of water was not included, because of its sensitivity to other motion (e.g. respiration) and its potential for pseudo/normalization following cerebral ischemia, e.g. [15]. For MR quantitation of ischemic changes, T_2_ values were measured in animals by an investigator blinded to group designation. Regions of interest were measured in several slices from each animal. The brain levels of interest included an MR slice containing the PT lesion, an MR slice immediately adjacent to but not containing a visible PT lesion, and an anterior slice remote from the lesion subjected to MCAO with the second insult (Fig. 1b). Measurements were made in the same slice levels after the first and second insult. Regions of interest in the right hemisphere included the temporal cortex (not directly affected by PT), the outer parietal cortex (i.e. the PT lesion in the PT slice), and mid-parietal cortex (peri-lesion region in the PT slice), along with proximate and adjacent regions of maximal intensity change near the PT lesion in the PT and adjacent slices, respectively. The PT lesion identified in the DW image 24 h post-PT was used to localize the lesion post-PT and on the corresponding slices post-tMCAO. Regions of homologous contralateral cortex were also measured.

### Histological analysis

At the time of euthanasia, rats were anesthetized with pentobarbital (80 mg/kg, IP), perfused with formalin, and brains were removed and embedded in paraffin. Sections (6 μm) were cut and stained with hematoxylin and eosin or Fluoro-Jade B (Histo-Chem, Inc., Jefferson, AR, USA), as described previously [13]. Altered staining or ischemic injury were assessed blinded to the animal’s surgical group. Cellular changes in sections stained with hematoxylin and eosin were sketched onto diagrams of coronal brain sections registering the MR and histology slices. The extent of cortical damage was identified by classifying regions of ischemic injury as selective necrosis, incomplete infarction, or pannecrosis [5].

### Statistical analysis

Data for the different experimental groups are reported as mean ± SD. Statistical comparisons were performed using SigmaPlot 13 software (Systat Software Inc., San Jose, CA, USA), which first tested for normalcy and equal variance of the data. Differences in means were considered significant at *P* < 0.05. Differences between ipsilateral and contralateral values were analyzed using a paired Student *t* test. Differences of means in sham + tMCAO and PT + tMCAO groups were analyzed using two-way analysis of variance (ANOVA). Differences in T_2_ relative to the temporal cortex were measured after PT and repeated after PT + tMCAO, and these were analyzed using a two-way repeated-measures ANOVA. As appropriate, this was followed by a Student–Neuman–Keul multiple comparison of means.

## Results

### Physiological measures

Groups were similar with regard to baseline measures such as body weight (Table 1). In addition, various physiological parameters measured or maintained during the insults, including rectal temperature and reductions in cortical perfusion during middle cerebral artery occlusion, did not differ between groups (Table 1).

**Table 1:** Mean values of physiological parameters measured in each of the experimental groups during the first surgery (sham or photothrombosis [PT]) or a subsequent transient middle cerebral artery occlusion (tMCAO)

	Experimental point	Control sham + tMCAO (*n* = 6)	PT + acute tMCAO (*n* = 6)	PT + chronic tMCAO (*n* = 5)
Weight (g)	Sham or PT	298 ± 48^a^	317 ± 25	270 ± 25
	tMCAO	309 ± 37	318 ± 25	322 ± 19
Temperature at end of the insult (°C)	Sham or PT	37.3 ± 0.1	37.5 ± 0.3	37.5 ± 0.1
	tMCAO	37.6 ± 0.3	37.6 ± 0.2	37.6 ± 0.1
Perfusion (% baseline)	During tMCAO	8.3 ± 2.8	7.8 ± 4.4	9.0 ± 2.4
	15 min post-tMCAO	142 ± 60	86 ± 35	101 ± 36

^a^Values shown are mean ± SD. Differences between groups n.s. (ANOVA)

### T_2_ imaging changes following the first photothrombotic insult

**Fig. 2:**
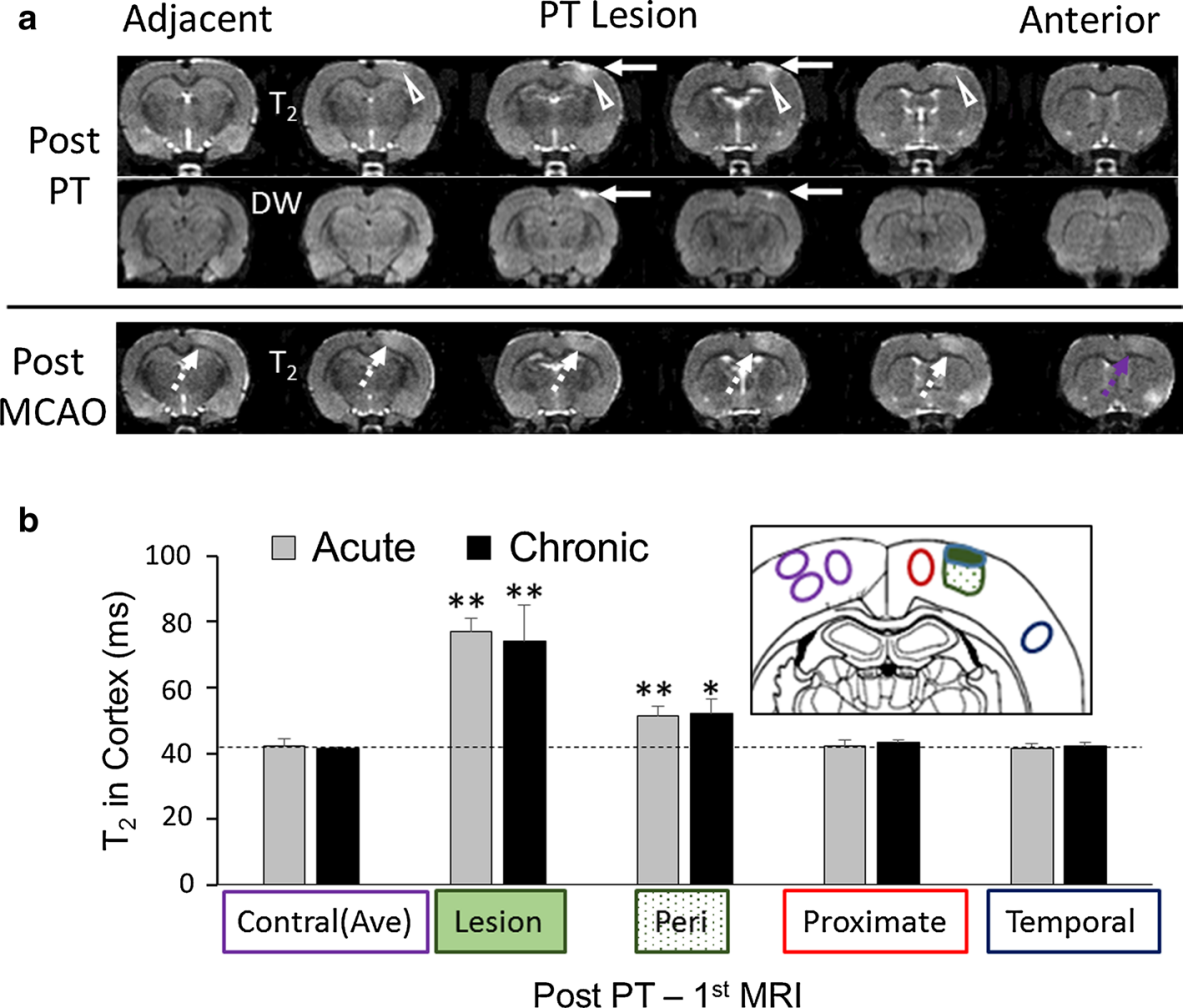
**a** MRI images from a representative rat in the acute group demonstrating T_2_ and DW intensity changes following a photothrombotic (PT) insult, and T_2_ changes subsequent to a transient middle cerebral artery occlusion (MCAO) 2 days later. Hyperintense T_2_ and DW changes (*arrows*) are observed within the PT lesion (a PT lesion slice level). Modest T_2_ changes associated with mild ischemic injury are observed in the peri-lesion region (*arrowheads*). Increased T_2_ in proximate lesion regions are apparent (*dashed arrows*). **b** Mean measures of T_2_ within cortical regions following PT in the acute and chronic groups. The schematic shows a representative location of ipsilateral regions of interest, including the PT lesion (*solid green*), peri-lesion (*stippled green*), proximate region (*red outline*) and temporal cortex (*blue outline*), along with homologous regions in the contralateral cortex. Ipsilateral increases in T_2_ in the PT region (paired Student’s *t* test, *P < 0.01; **P < 0.005 different from contralateral) are similar between the acute and chronic groups (Student’s *t* test, *P* > 0.2)

As expected for ischemic lesions produced using the PT model [13], inspection of the MR images acquired 1 day following PT demonstrated the presence of cortical ischemic lesions within several MR slices consisting of an outer cortical lesion of hyperintense DW increase corresponding to an area of marked T_2_ hyperintensity (e.g. arrows, Fig. 2a). T_2_ increases were maximal in middle slices, corresponding to brighter illumination centrally. In addition, a peri-lesion region of a more modest T_2_ increase (e.g. arrowheads, Fig. 2a) was observed. The mean areas of DW hyperintensity in the middle PT slice were of a minor size and similar in magnitude between the acute and chronic groups (0.62 ± 0.26 and 1.02 ± 0.62 mm^2^, respectively). Quantitative measures of T_2_ demonstrate that, following the PT procedure, there was an increased T_2_ in both the lesion and peri-lesion cortex compared to T_2_ in the homologous contralateral control cortex (Fig. 2b). The ischemic T_2_ changes in the PT lesion were similar irrespective of whether the animals were in the acute or chronic groups, indicating a similar severity of the first ischemic insult.

### Effect of tMCAO in sham controls

**Fig. 3:**
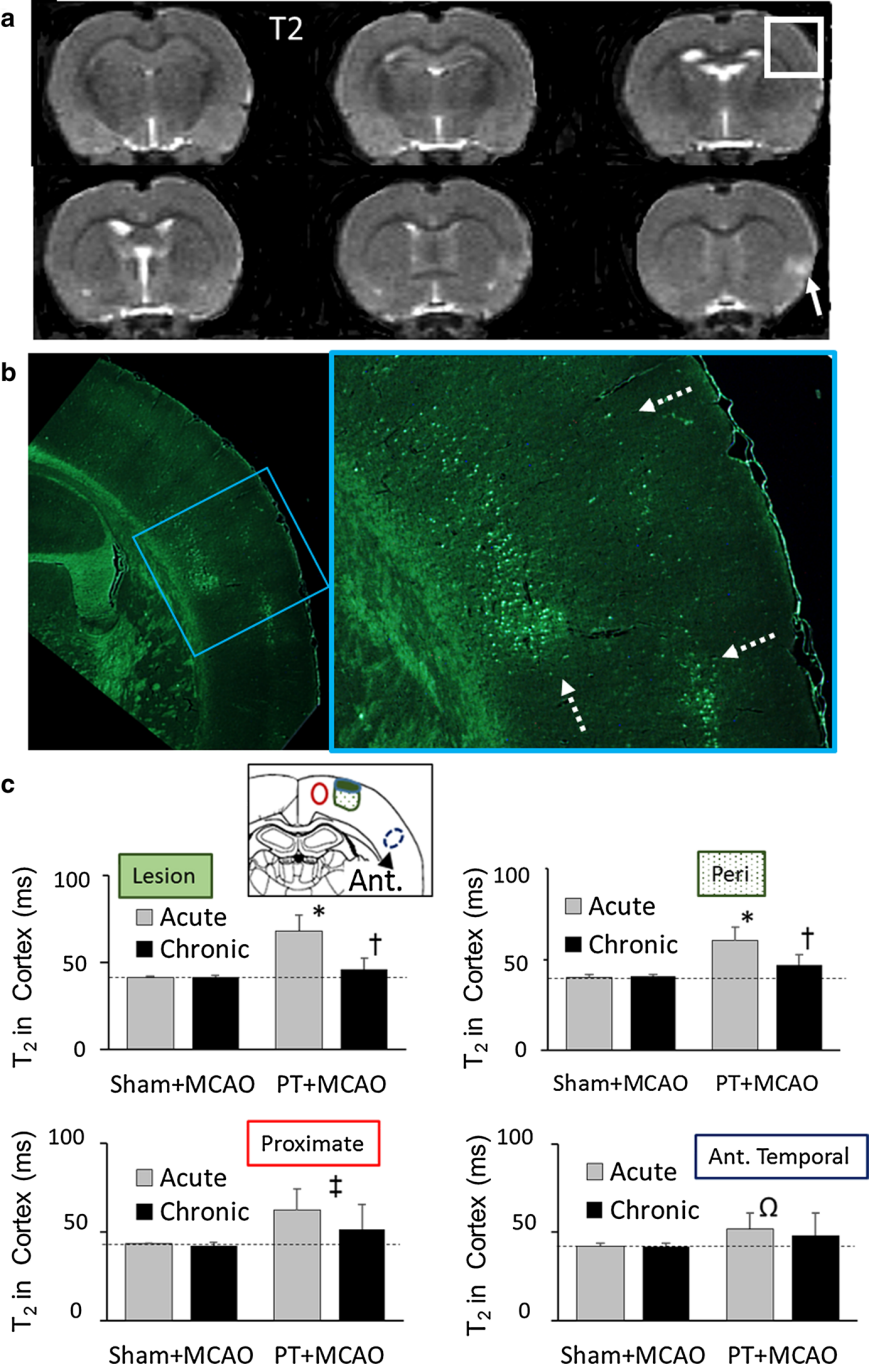
Effect of right transient middle cerebral artery occlusion (MCAO) in the sham control group. **a** Six T_2_-weighted slices from an animal in the sham control group 1 day following MCAO. An increase in T_2_ is observed at the site of clip MCAO (*arrow*) but no apparent MR change in the MCA territory. **b** Corresponding histological sections (high and low magnification) of a cerebral cortex region (*white box* in MR images) stained with Fluoro-Jade. Scattered labeled dead cells (*dashed arrows*) are present throughout the MCA territory. Normal white matter has endogenous fluorescence. **c** T_2_ changes in four of the regions of interest (shown in the schematic) for the groups with a sham or PT procedure followed by an acute or chronic recovery time prior to MCAO. Details of the two-way ANOVA are presented in Supplementary File 2. Within the lesion and peri-lesion regions, there are increases in T_2_ following PT with acute tMCAO (*P < 0.001, PT + MCAO different from sham + MCAO within the acute groups). Also, within the lesion and peri-lesion regions, in the PT + MCAO group there is a difference depending on the recovery time prior to tMCAO (^†^ *P* < 0.005, acute different from chronic). Within the proximate region, there is an increase in T_2_ following tMCAO, irrespective of recovery time between insults (^‡^ *P* < 0.03 PT + MCAO different from SH + MCAO, chronic vs. acute, n.s., power suboptimal). In the temporal cortex of an anterior (Ant) slice, there are no significant differences between groups (power suboptimal), although there is an ischemic ipsilateral increase in the temporal cortex in the acute PT + MCAO group (^Ω^ *P* < 0.05; ipsilateral different from contralateral, paired Student’s *t* test)

In animals with a sham PT procedure followed by tMCAO, cortical changes in T_2_ were not apparent in comparison to the contralateral hemisphere (e.g. Fig. 3a); there was often an increase in T_2_ in the cortex near the microclip placement (arrow, Fig. 3a), and this area was avoided for quantitation. Furthermore, mean T_2_ within the anterior temporal cortex supplied by the middle cerebral artery was normal after a sham surgery followed by tMCAO (e.g. 41.3 ± 2.4, 40.7 ± 2.4 and 40.5 ± 1.2 in the sham + acute MCAO, sham + chronic MCAO and post-PT [no MCAO] groups, respectively). Despite a lack of apparent T_2_ changes, there was evidence in the hematoxylin and eosin or Fluoro-Jade-stained sections of mild ischemic changes within the MCA territory visible as scattered cell death in all six of the control animals (e.g. Fig. 3b).

### T_2_ Changes with PT followed by a second insult of tMCAO

**Fig. 4:**
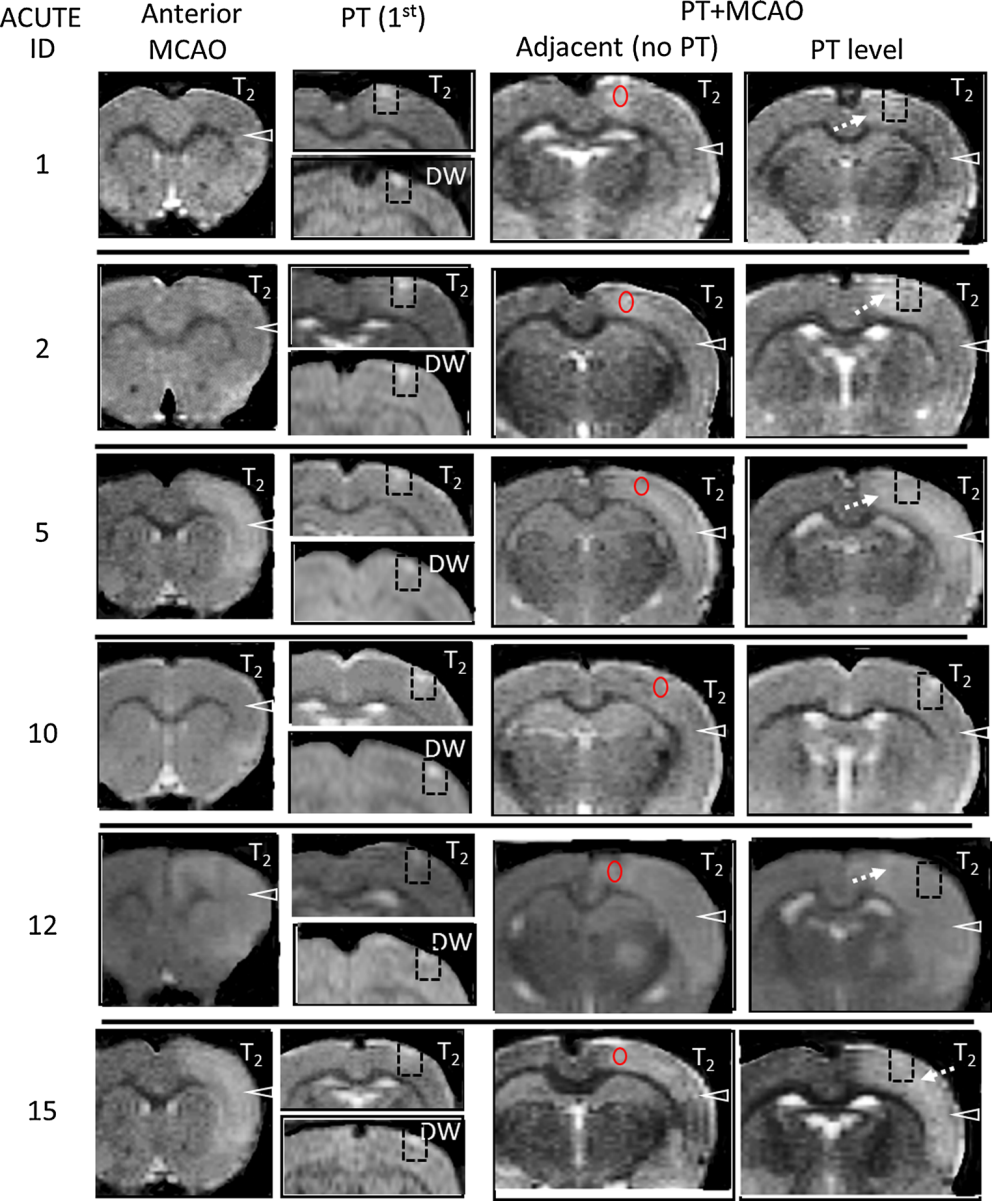
MRI scans from the levels used for quantitation for each of the animals in the acute group. Following PT, the region (*dashed box*) that includes the PT lesion (i.e. the DW hyperintense area) and a peri-lesion region has increases in T_2_ above that in homologous contralateral cortex. These changes partially resolved post-transient middle cerebral artery occlusion (tMCAO). Following tMCAO, in the PT level, a proximate lesion area (e.g. *dashed arrows*) is often hyperintense relative to that in the temporal remote cortex (*arrowhead*), as are regions adjacent to the lesion (*outlined in red*) in the slice adjacent to the PT

**Fig. 5:**
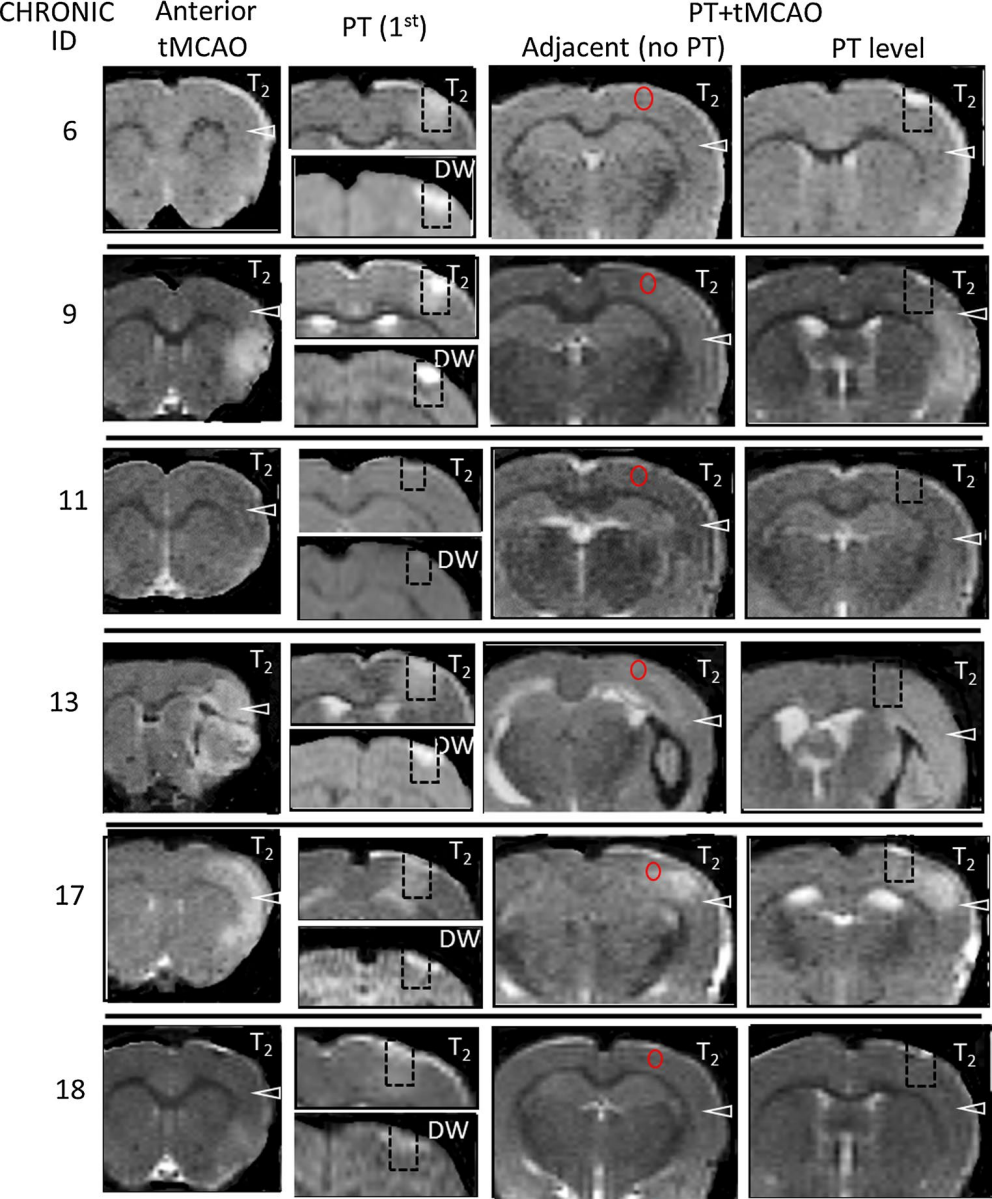
MRI scans from the levels used for quantitation for each of the animals in the chronic group. Within the *dashed box*, the PT lesion area (the DW hyperintense area) and the peri-lesion cortex have increased T_2_ intensity, which tend to resolve post-transient middle cerebral artery occlusion (tMCAO). Following tMCAO, T_2_ intensity in peri-lesion and proximate areas and regions adjacent to the PT in the adjacent slice (*outlined in red*) are similar or somewhat higher than that in the temporal cortex (*arrowhead*). Animal 13 had an intracerebral hemorrhage and was excluded from the quantitative analysis

In contrast to the sham + tMCAO controls, the majority (4/6) of animals in the acute PT + tMCAO group had substantial increases in T_2_ (11, 34, 50, and 51% of contralateral) within the anterior temporal cortex of the MCA territory (MRI for each rat shown in Fig. 4). In the chronic PT + tMCAO group (Fig. 5), increases in T_2_ of 14, 40, and 74% of contralateral were observed in 3/6 animals, and the last of these animals (ID 13) had a substantial intracerebral hemorrhage. This outlier was excluded from further quantitative analysis. Thus, consistent with our previous study [5], there was a significant mean ipsilateral versus contralateral increase in T_2_ (*P* < 0.05) within the anterior MCA territory following tMCAO in the acute group that was not present in the chronic group (Fig. 3c). The direct comparison of T_2_ within the anterior temporal cortex for the acute and chronic sham + MCAO and PT + MCAO groups was not significant; however, power was suboptimal for these sample sizes and variations in response to tMCAO.

Within the PT lesion and perilesion regions, there were differences between the sham + tMCAO and PT + tMCAO groups (Fig. 3c). The PT lesion and peri-lesion regions with an acute recovery prior to tMCAO had greater T_2_ values than with chronic recovery. However, these changes are likely affected both by the known normalization over time of the ischemic T_2_ increase that occurs following PT [13] in addition to the effects of tMCAO. Importantly, in a region of maximal T_2_ change proximate to the PT lesion (i.e. without confounding effects of initial PT changes) there was also a significant increase in T_2_ in the PT + tMCAO group compared to the sham + tMCAO groups; and, despite a trend for differences this was not dependent on whether recovery was acute or chronic prior to the MCAO (power suboptimal).

**Fig. 6:**
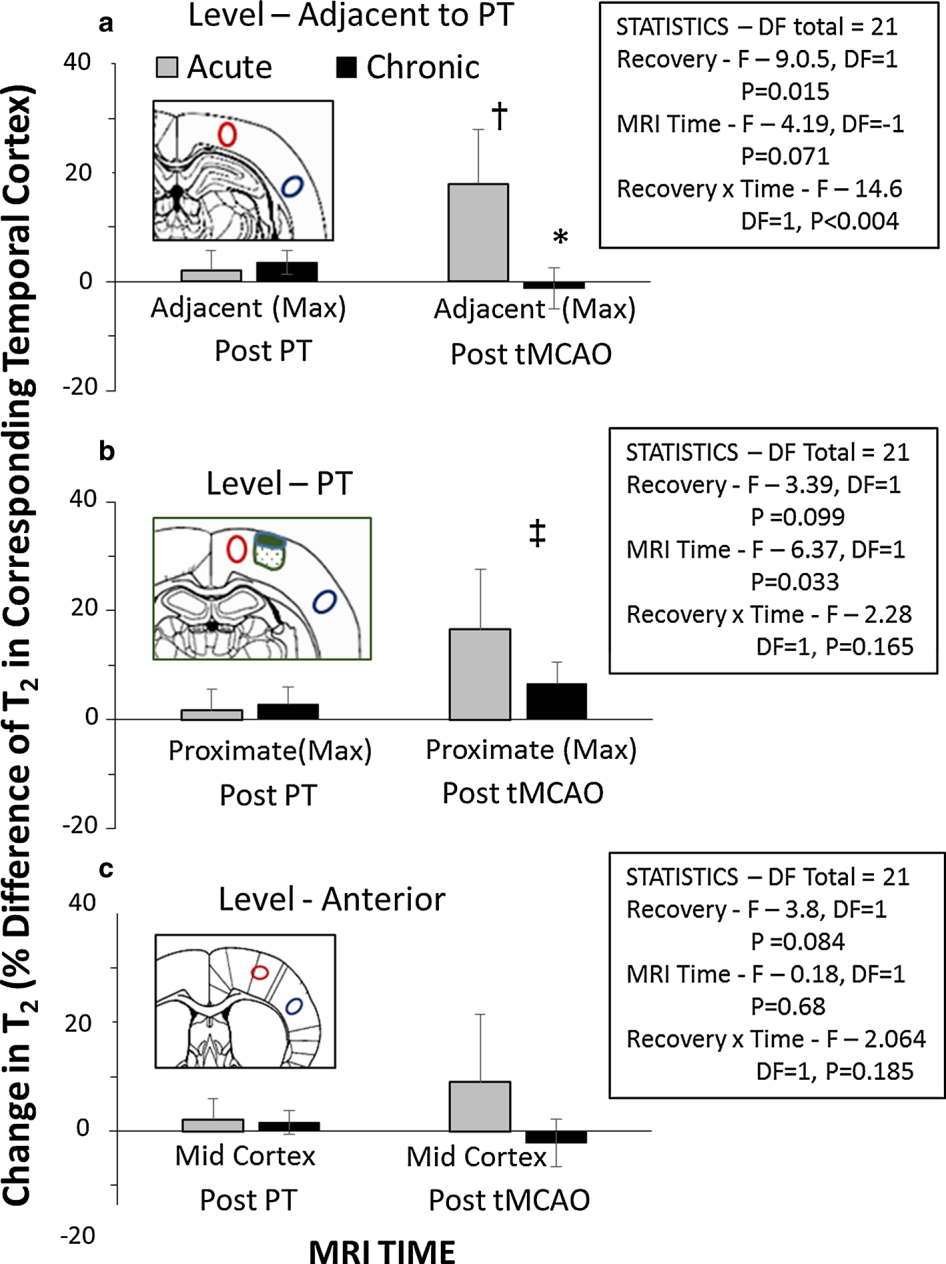
Quantitation of T_2_ changes within regions initially unaffected by photothrombosis (PT) but often displaying increased MR intensity following transient middle cerebral artery occlusion (tMCAO) at acute or chronic recovery. Regions of interest were located in coronal levels **a** adjacent to the PT, **b** within the PT, and **c** anterior to the PT. Schematics show representative locations of regions including the PT lesion and peri-lesion areas (*green solid and stippled* areas, respectively), the proximate and adjacent region of interest near the PT in the PT and adjacent slices, respectively (maximal changes *outlined in red*), and a region within corresponding temporal cortex (*blue outline*). Details of the two-way repeated measures ANOVA are shown in the boxes. Within the adjacent level (**a)**, the change in T_2_ in the cortex adjacent to the lesion (as a percentage of temporal cortex) differed between the acute and chronic groups at the post-tMCAO MRI time (*P < 0.001, Student–Neuman–Keul multiple comparison of means). Also, the T_2_ changes in the acute recovery group differed between the post-PT and post-tMCAO MRI time (^†^ *P* < 0.002). For the PT level (**b**), post-MCAO differed from post-PT (^‡^ *P* < 0.04) in the proximate region, but the trend for dependence on whether recovery was acute or chronic was not significant (power suboptimal). In the anterior level (**c**) remote from the PT, T_2_ within the cerebral cortex was rather homogeneous in the parietal and temporal cortex (comparisons n.s; statistical power suboptimal)

In order to investigate the contribution of T_2_ changes due to tMCAO, MRI changes within regions near but unaffected by the initial PT were selected for further analysis (i.e. using the MRI measures post-PT and post-tMCAO, data in Supplementary File 1). In addition, alterations in the regions of interest were normalized to the T_2_ changes in the temporal cortex for each animal (Fig. 6). Within the MR slice adjacent to the PT (Fig. 6a), there was a T_2_ increase in the region adjacent to the PT after tMCAO (presented as a percentage of that in the temporal cortex; *P* < 0.005), which was significantly greater in the acute versus chronic groups (*P* < 0.001). The differences in T_2_ in the acute PT + tMCAO group were similar if animals with (*n* = 4) and without (*n* = 2) temporal T_2_ changes were averaged separately (i.e. 16.8 ± 5% and 20.3 ± 16%, respectively). Within an MR slice at the PT level (Fig. 6b), there was an increase in T_2_ within a region proximate to the PT after tMCAO, irrespective of whether the recovery was acute or chronic (*P* < 0.04). Although the interaction between factors was not significant at this level, a lack of effect of recovery needs to be interpreted cautiously, as power was suboptimal. Within the anterior slice level, there were no significant T_2_ increases, both because this slice level was remote from the PT and because, following tMCAO, the T_2_ increases in the cortex were rather homogeneous—i.e. similar to those in the temporal cortex. However, again power was suboptimal.

### Histological correspondence of the combined insults

Brain damage detected in Fluoro-Jade and hematoxylin and eosin-stained sections generally corroborated the T_2_ changes observed. When MCAO produced substantial ischemic T_2_ changes there was visible incomplete infarction or pannecrosis within the temporal cortex of the MCA territory on hematoxylin and eosin-stained sections and diffuse high fluorescence in Fluoro-Jade-stained sections.

**Fig. 7:**
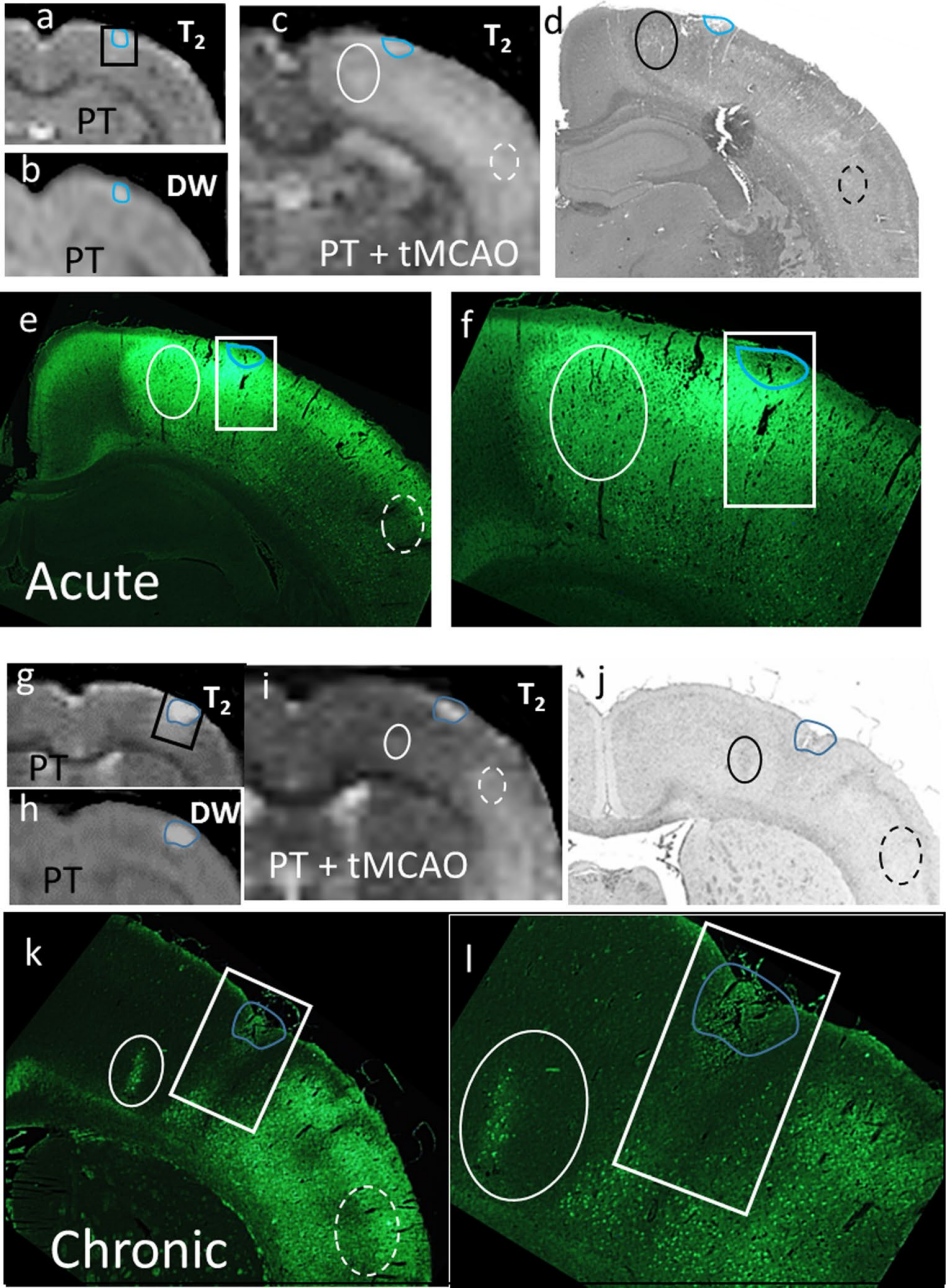
Example of histological changes in an animal from the acute (**a**–**f**) and chronic (**g**–**l**) groups for which T_2_ was increased in the MCA territory post-MCAO. The MRI changes with PT (**a**, **b**, **g**, **h**) produced minor ischemic hyperintense lesions *outlined in blue* in DW images that are copied onto T_2_ and histological images. The *rectangle* encloses the region of hyperintense T_2_ change post the initial PT. The lesion region is shown in subsequent MR (**c**, **i**), hematoxylin and eosin (**d**, **j**), and Fluoro-Jade images (×2—**e**, **k** and ×4 **f**, **l** magnification). Maximal proximate T_2_ changes (e.g. *solid oval*) were prominent in the acute but not chronic animals compared to corresponding changes in the temporal cortex (*dashed oval*). In the acute animal, this corresponded to an area of marked positive Fluoro-Jade fluorescence and increased vacuolation in the hematoxylin and eosin-stained sections. In the chronic animal, such a proximate region had less positive Fluoro-Jade staining than in the temporal cortex (**k**, **l**)

In animals with temporal T_2_ increases following PT + tMCAO, in the acute group, regions in the vicinity of the lesion with increased T_2_ generally corresponded to increased Fluoro-Jade staining and marked vacuolation in the hematoxylin and eosin-stained sections (e.g. Fig. 7a–f). In contrast, in the chronic group, peri-lesion and proximate regions tended to have a lack of both T_2_ augmentation and Fluoro-Jade staining compared to remote temporal cortex, suggestive of less damage near the lesion when there was chronic recovery between insults (e.g. Fig. 7g–l).

**Fig. 8:**
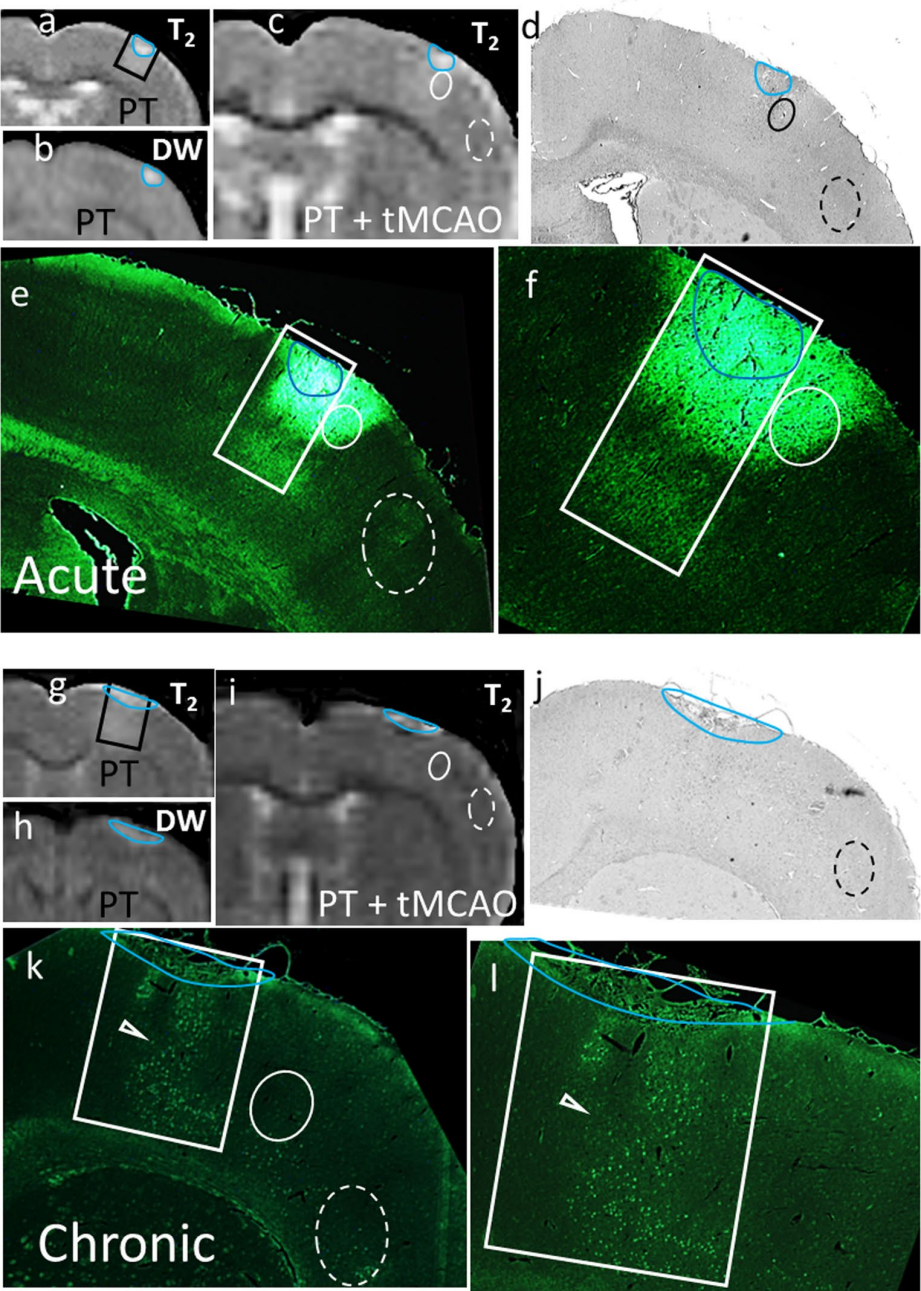
Example of histological changes in an animal from the acute (**a**–**f**) and chronic (**g**–**l**) groups for which T_2_ was not increased in the MCA territory post tMCAO. The MRI changes following PT (**a**, **b**, **g**, **h**) produced ischemic hyperintense lesions outlined in blue in DW images that were copied onto T_2_ and histological images. The rectangle encloses the region of hyperintense T_2_ change post the initial PT. The lesion region is shown within subsequent MR (**c**, **i**), hematoxylin and eosin (**d**, **j**) and Fluoro-Jade images (×2—**e**, **k** and ×4 **f**, **l** magnification). Regions proximate to the lesion (e.g. *solid oval*), with enhanced T_2_ changes relative to changes in temporal cortex post-tMCAO (*dashed oval*), are observed in the Acute animal but not in the chronic animal. In the acute animal, this corresponded to an area (solid oval) of marked positive Fluoro-Jade fluorescence (**e**–**f**). In the chronic animal, the peri-lesion region had scattered positive Fluoro-Jade-stained cells within the region of increased T_2_ observed post-PT (*arrowhead*, **k**, **l**) and the remote temporal cortex had scattered cell necrosis as expected following mild tMCAO

In animals with PT + tMCAO and no obvious T_2_ change within the MCA territory, there was scattered cell death in the MCA territory in both groups (e.g. Fig. 8). In the acute group, there was also apparent increased T_2_ in proximate regions corresponding to increased Fluoro-Jade staining (e.g. Fig. 8a–f). In chronic animals (e.g. Fig. 8g–l), the greatest density of scattered cell death was usually observed within the peri-PT lesion region. Although this is consistent with the injury associated with increased T_2_ after the initial PT insult, it is possible that some of these dying cells were produced by ischemia during the tMCAO. Cell death in proximate regions in chronic animals was often similar to that in the temporal cortex.

## Discussion

In the current study, we report original results regarding the MRI detection of the combined injury produced by a mild ischemic insult within regions near an initial minor stroke. To the best of our knowledge, our results are the first to demonstrate that (1) T_2_ MRI can detect additional injury resulting from the combination of a model of minor stroke and diffuse cortical transient ischemia in the adult brain, (2) regions proximate to a small ischemic lesion may depict pronounced damage or MRI changes as a result of a second ischemic event, and (3) in contrast to acute recovery, with a longer *chronic* recovery of 1 week between insults, there was a lack of MRI and histological evidence for augmentation of brain injury in MRI slices and areas adjacent to the first minor stroke.

The mild ischemic injury that was seen in the current study, which consisted of scattered necrosis produced by tMCAO following a sham PT surgery, is similar to that observed previously [5]. A PT followed acutely by tMCAO frequently resulted in substantial ischemic injury within the cortex in the MCA territory, including the temporal cortex. This is also consistent with a previous study in mild recurrent stroke [5] that used multiple tMCAO producing two episodes of mild ischemia throughout the middle cerebral artery cortex territory, and damage throughout the cortex was enhanced when recurrence was acute (1 day) compared to subacute (3 days). However, with this previous protocol design, it was difficult to assess the source of differences in recurrence severity, which were possibly a combination of evolving effects of an initial ischemic injury—either systemic or cerebral. Indeed, there were data supporting cerebral differences between 1 and 3 days (e.g. in microglial activation), but also evidence for systemic differences between groups, such as increased granulocytes and decreased platelet levels in the blood at the acute compared to subacute time points. In the current study, the more focal initial PT procedure appeared to add variability to the severity of ischemia produced by tMCAO alone, perhaps by altering overall cerebrovascular resistance and collateral flow patterns or by adverse systemic responses in some animals. Such variability contributed to suboptimal power for some of the statistical calculations performed, and a limitation of the current study was the small sample sizes. Future experiments should be repeated with larger numbers to determine the reproducibility of the findings, particularly for results where power was suboptimal. Sub-stratification of recovery times and the use of larger sample sizes to allow for sub-analysis of animals grouped according to whether mild tMCAO produced MRI changes would also be desirable. Ideally, such a study would have a comprehensive experimental design that includes complete groups with MRI following the sham PT prior to the acute or chronic tMCAO, and groups with PT followed by acute or chronic sham, including MRI after each procedure.

Despite these shortcomings, a key advantage of the current experimental design was its use of repeated MRI following the insults to localize the effects of combined injury. The first MRI identified the location and severity of the minor stroke and its peri-lesion area of modest T_2_ increases. The repeat MRI following the second, more diffuse cortical transient ischemia was used to directly measure the effect of tMCAO on T_2_ in the proximity of the lesion. To avoid the confounding effects of the normalization of T_2_ hyperintensities following PT, we focused on the imaging changes in regions not affected by the first PT insult. The results showed that in the proximate lesion region within the PT MRI slice, there were rather consistent increases in T_2_ that exceeded those in the temporal cortex in the MCA territory. Such enhancement extended to an adjacent lesion region in an adjacent MR slice when there was an acute but not chronic recovery time between insults. Heterogeneous increases in T_2_ within the vicinity of a PT, generally consistent with those of the present study, have also been observed in neonatal rats subjected to a minor PT followed 2 days later by unilateral cerebral hypoxia–ischemia [16].

The cause of augmented regions of ischemic injury in the proximity of a minor PT is unknown. Local tissue injury produced by the first minor stroke soon after transient ischemia may be a contributing factor. However, the hyperintense T_2_ regions adjacent to the initial PT lesion had variable localization that was not within the initial peri-infarct region identified by modest T_2_ changes. Also, within slices containing the PT, an increased T_2_ above that within the temporal cortex was observed in both the chronic and acute PT + tMCAO groups. This suggests that other factors such as variations in vascular anatomy along with PT location may perturb the regional ischemic severity of the second tMCAO, thereby enhancing the damage. Indeed, single or multiple arteriolar occlusions, depending on their location, have complex effects on collateral flow and ischemic severity [17].

The reason for a lack of increased T_2_ and histological damage within the adjacent MRI slice when recovery was chronic is also uncertain. It is possible that during recovery from the first PT, plasticity and repair processes are activated in the vicinity of the PT, or that there is development of additional vascular collaterals around the minor infarct, which could reduce damage by improving local perfusion during the second ischemic episode [18, 19]. Indeed, several studies have investigated plasticity and repair post-PT demonstrating the elevation of factors 1 week post-PT, such as vascular endothelial growth factor, CD11 staining of microglia, or nerve growth factor-induced gene-B mRNA [20–22]. With regard to collateral development, cortical blood flow measures following a PT ring insult indicate that cortical perfusion is reduced over the first few days, followed by normalization of blood flow over 3–7 days—an observation proposed to be due to the development of collaterals or recanalization [23–25]. Our current Doppler flow measures in the cortex did not detect differences between groups in flow reduction in the parietal cortex during tMCAO, but these values are relative to baseline and are also unable to identify regional flow differences prior to MCAO. Histological evidence for angiogenesis is provided in a study using BrdU and VEGF staining to indicate that, following a PT, there is pronounced angiogenesis in the cortex by 3 days, continuing well past 1 week [20]. Future studies will be needed to identify which factors are involved in mediating the different responses to recurrent stroke observed adjacent to the lesion.

Although augmented damage was detected by T_2_ imaging in the present study, there are limitations regarding its use for diagnosing cumulative damage. The timing of the imaging is important, and appears to be optimal if performed acutely, e.g. 24 h post-PT, because T_2_ normalizes as tissue edema resolves. Also, T_2_ MRI detected the effect of combined injury once it was relatively severe, consisting of partial or complete infarction. T_2_ imaging was not sensitive to the detection of scattered cell death in the cortex following transient MCAO. Thus, detection of mild ischemic injury remains a challenge. Positron emission tomography (PET) imaging with ^11^C-flumazenil has been used to demonstrate the presence of selective neuronal loss in the non-infarcted penumbra post-stroke [11, 12, 26], but such sophisticated PET technology is not yet widely available or routinely used clinically. The present results indicate there are complex interactions between mild ischemic insults regionally, which calls for additional research to improve the imaging detection of selective necrosis. Nevertheless, the current findings provide an important basic step toward developing approaches for improved detection, management, and treatment of such recurrent cerebrovascular insults.

## Conclusions

Within a region proximate to an experimental minor stroke, an acute (1–2 days) or chronic (7 days) recurrent mild transient cerebral ischemia was found to augment T_2_ above increases produced by tMCAO alone, reflecting increased tissue damage in this region. Augmented ischemic injury detected adjacent to the lesion extended to adjacent slices when recovery between insults was acute but not chronic.

## Electronic supplementary material

Below is the link to the electronic supplementary material.

Supplementary material 1 (DOCX 13 kb)

Supplementary material 2 (XLSX 15 kb)
