# Supplementary material for: Magnetic resonance imaging detection of multiple ischemic injury produced in an adult rat model of minor stroke followed by mild transient cerebral ischemia
Source: MAGMA. 2016 Nov 4;30(2):175–88. doi: 10.1007/s10334-016-0597-5 (PMC5364243; doi:10.1007/s10334-016-0597-5)
Supplement: Supplementary file 1 — Supplementary material 1 (DOCX 13 kb) [file 10334_2016_597_MOESM1_ESM.docx]

Table Statistical Details of the Two Way Analysis of Variance of the T_2_ Measures for the Regions* presented in Figure 4c. Independent Variables analyzed were Recovery Time between insults (Acute or Chronic) and Insult Type (Sham or PT) prior to transient middle cerebral artery occlusion (MCAO)*

| Region | First Insult Type:  Sham or PT | | Recovery prior to MCAO:  Acute or Chronic | | Interaction  InsultxRecovery | |
| --- | --- | --- | --- | --- | --- | --- |
|  | F Statistic | P value | F Statistic | P value | F-Statistic | P value |
| PT Lesion | 17.3 | 0.001 | 8.56 | 0.012 | 8.77 | 0.011 |
| PT Peri | 16.9 | 0.001 | 4.14 | 0.063 | 4.93 | 0.045 |
| PT Adjacent | 5.95 | 0.03 | 0.83 | 0.38 | 0.71 | 0.42 |
| Ant. Temporal | 2.08 | .17 | 0.19 | 0.67 | 0.09 | 0.77 |

*Groups: Sham+Acute MCAO, Sham+Chronic MCAO (n=3/group), PT+acute MCAO (n=6) or PT+chronic MCAO (n=5). Degrees of freedom: total of 16 and 1 for each variable.
